# Supplementary material for: Targeted Metabolomics Study on the Effect of Vinegar Processing on the Chemical Changes and Antioxidant Activity of Angelica sinensis
Source: Antioxidants (Basel). 2023 Nov 28;12(12):2053. doi: 10.3390/antiox12122053 (PMC10740601; doi:10.3390/antiox12122053)
Supplement: Supplementary file 1 [file antioxidants-12-02053-s001.zip › antioxidants-2720559-supplementary.pdf]

## Supporting information

| No. | Content |
|---|---|
| Figure S1 | TIC overlaps of quality control samples and XIC of multi-peak detection plots of metabolites. TIC by mass spectrometry detection (A, B) and XIC in the MRM mode (C, D). A and C were acquired in positive ionization mode. B and D were acquired in negative ionization mode. |
| Figure S2 | PCA scores plot of quality spectrum data in each group. |
| Figure S3 | Score plot, S-plot, and verification diagram of OPLS-DA. OPLS-DA score plot (A), OPLS-DA S-plot (B), and OPLS-DA verification diagram (C) of different groups. |
| Figure S4 | The relative contents of the typical compounds in A, S-A, and V-A. |
| Figure S5 | Plots of clustering heatmaps, dynamic distribution, and radar map analysis between A and S-A. Clustering heatmaps (A), dynamic distribution (B), and radar map (C). |
| Figure S6 | Plots of clustering heatmaps, dynamic distribution, and radar map analysis between A and V-A. Clustering heatmaps (A), dynamic distribution (B), and radar map (C). |
| Figure S7 | Cluster heat map analysis of various differential compounds. |
| Table S1 | Sample information collection sheet. *A. Sinensis* sample number, plant Origin, Site of Tissue, processed sample and group. |
| Table S2 | 83 differential compounds of *A. sinensis* during processing. |
| Table S3 | Grey correlation degree analysis. Grey correlation degree analysis between differential components and antioxidant activity of *A. sinensis* and its processed products. |

**Figure S1. TIC overlaps of quality control samples and XIC of multi-peak detection plots of metabolites. TIC by mass spectrometry detection (A, B) and XIC in the MRM mode (C, D). A and C were acquired in positive ionization mode. B and D were acquired in negative ionization mode.**

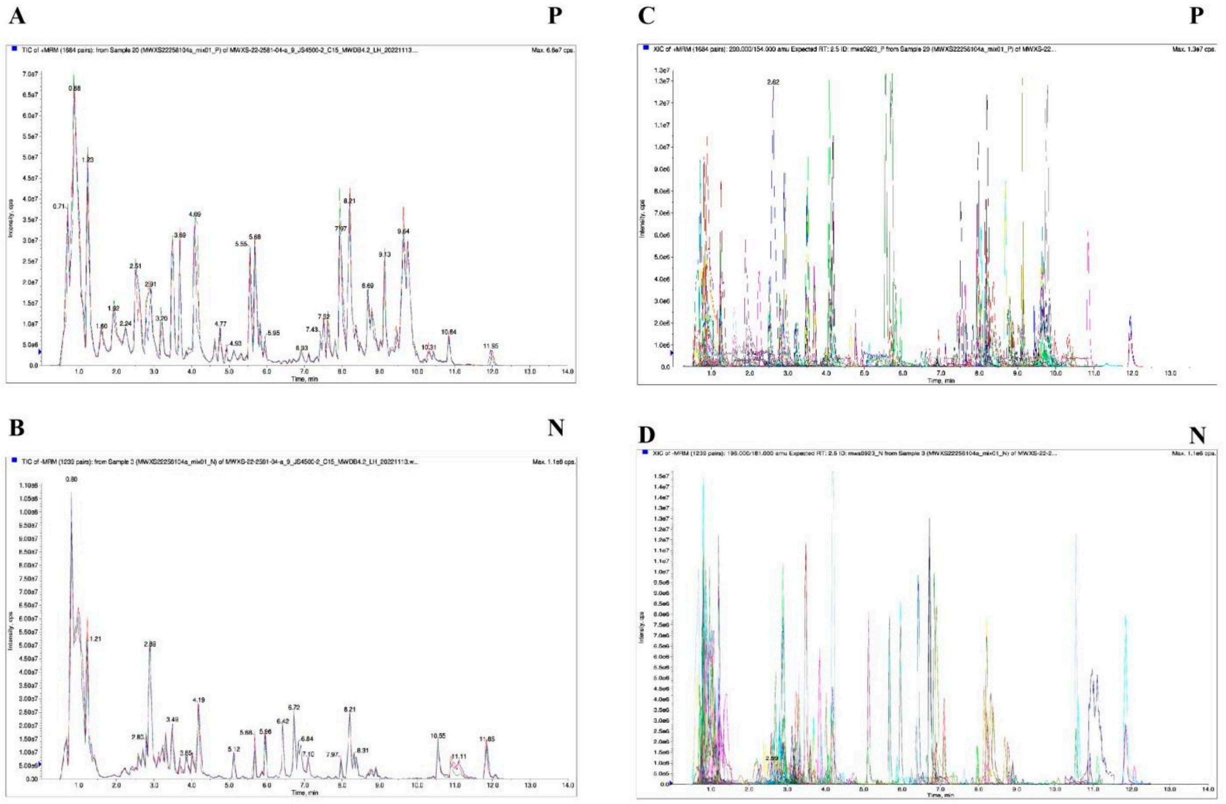

**Figure S2. PCA scores plot of quality spectrum data.**

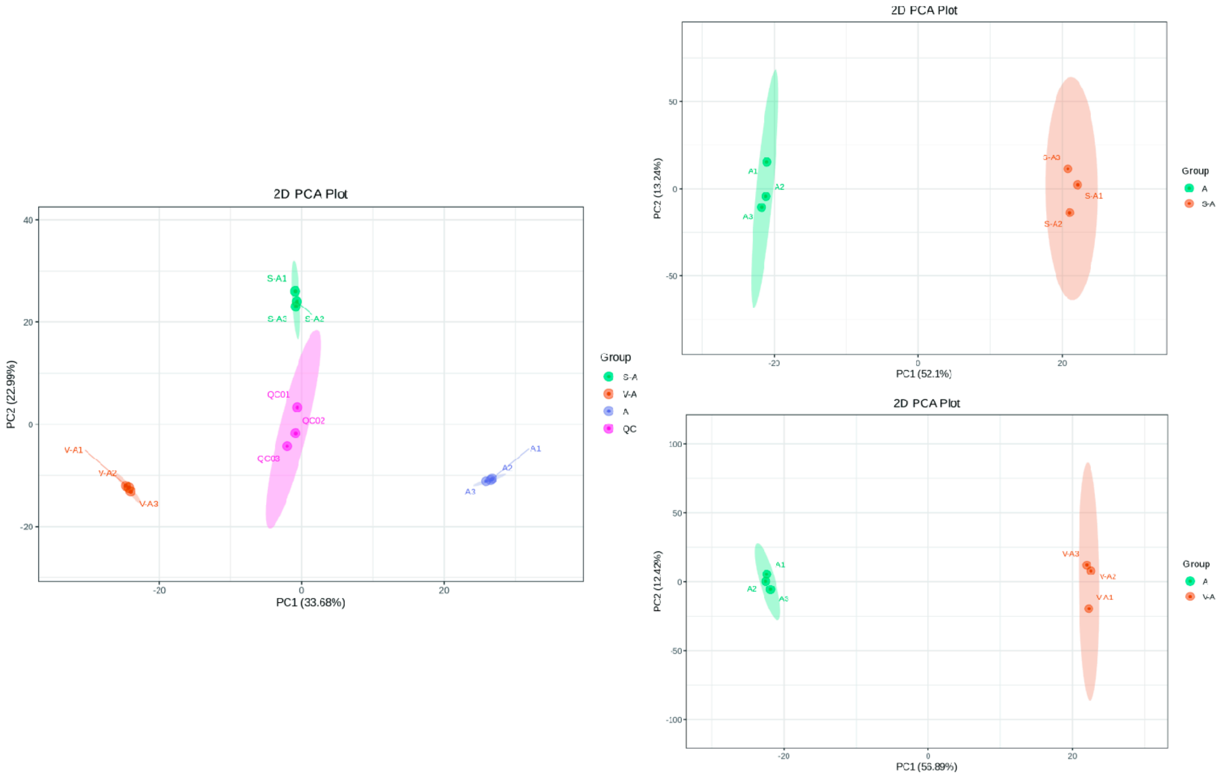

**Figure S3. Score plot, S-plot, and verification diagram of OPLS-DA. OPLS-DA score plot (A), OPLS-DA S-plot (B), and OPLS-DA verification diagram (C) of different groups.**

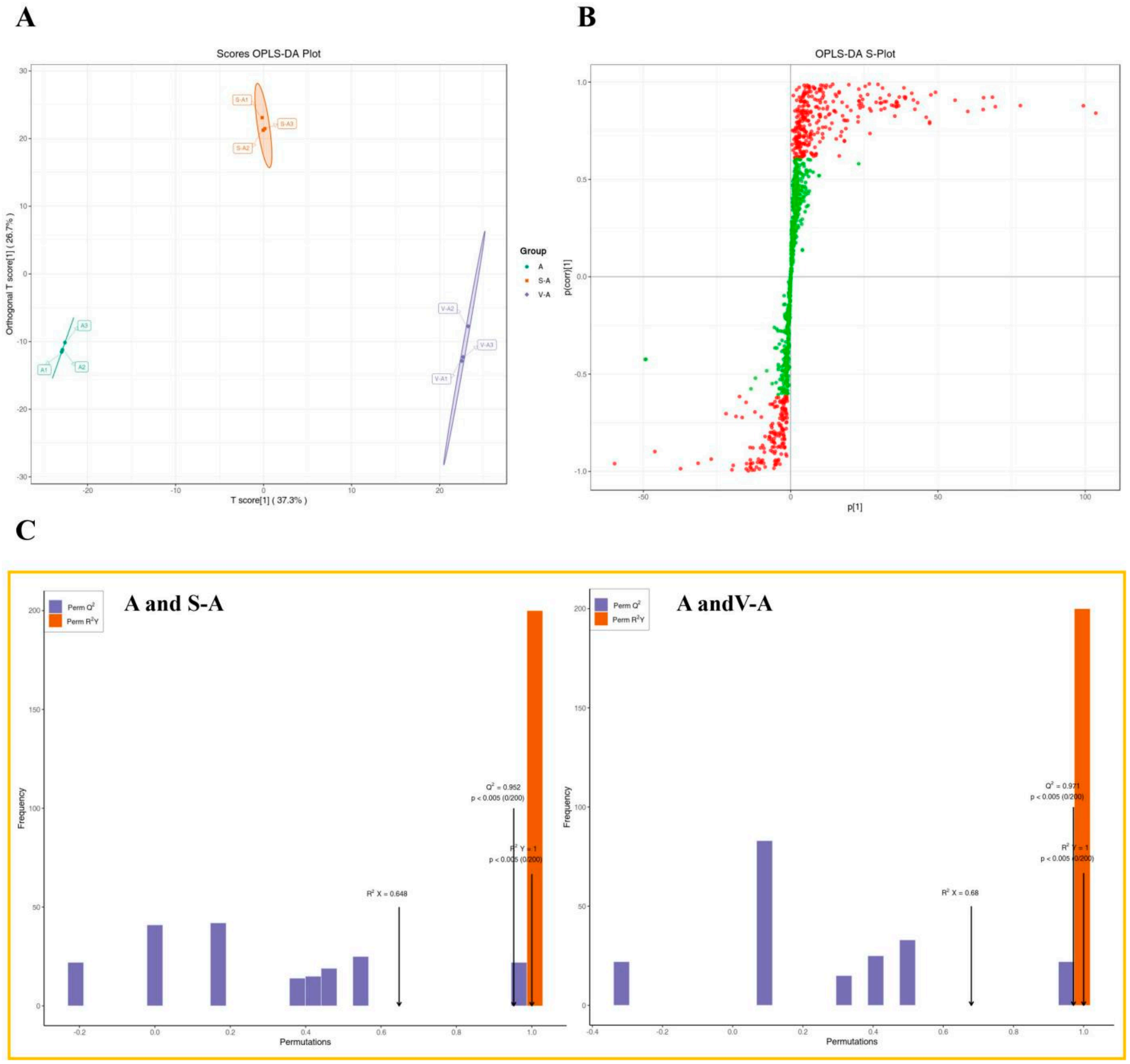

**Figure S4. The relative contents of the typical compounds in A, S-A, and V-A..**

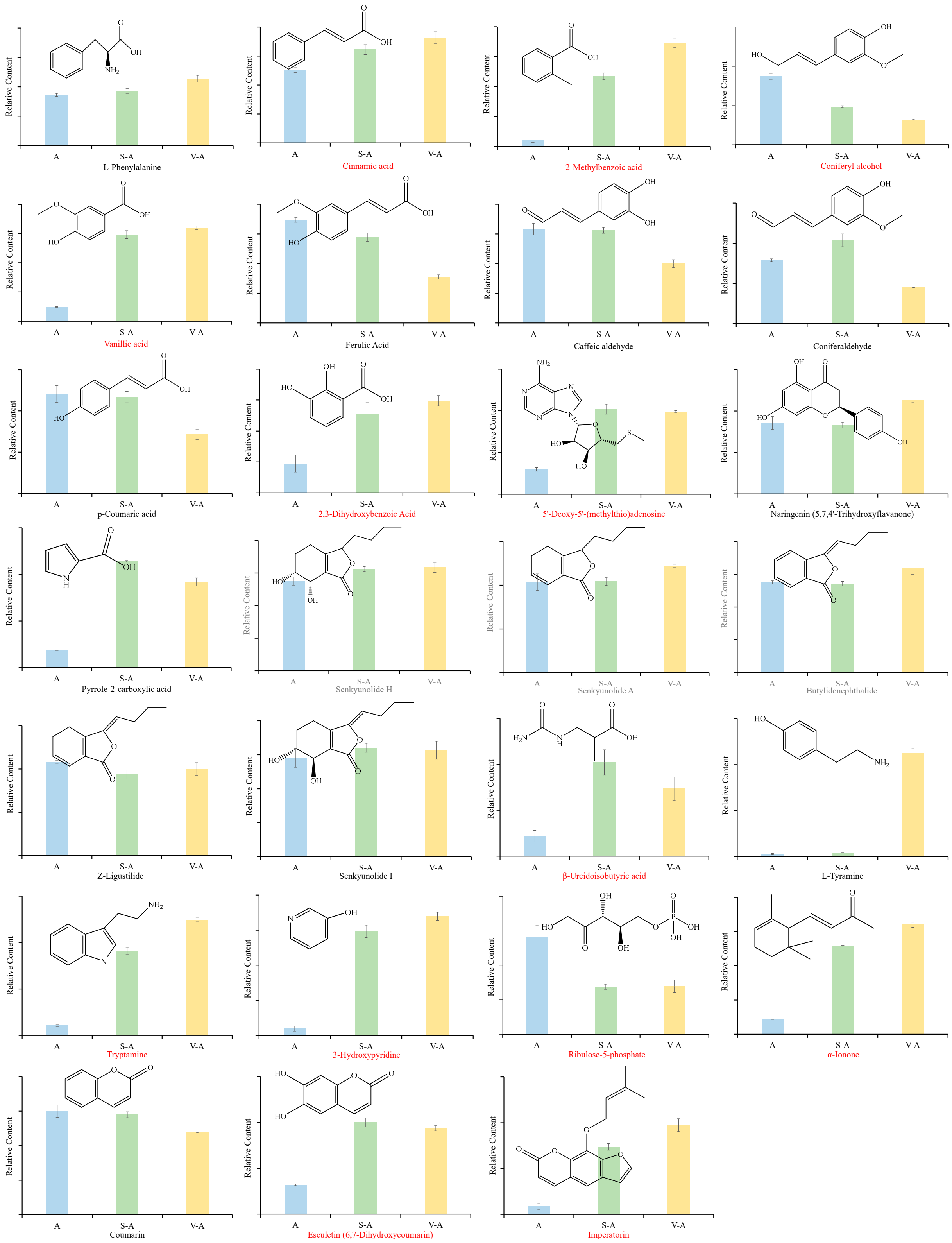

**Figure S5. Plots of clustering heatmaps, dynamic distribution, and radar map analysis between A and S-A. Clustering heatmaps (A), dynamic distribution (B), and radar map (C)..**

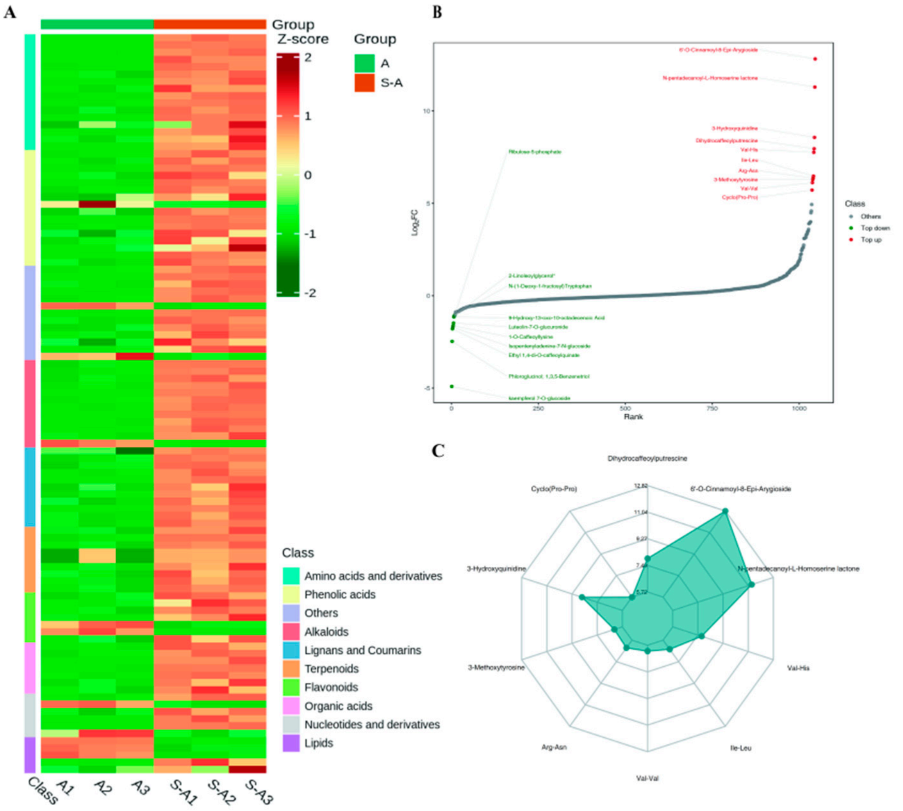

**Figure S6. Plots of clustering heatmaps, dynamic distribution, and radar map analysis between A and V-A. Clustering heatmaps (A), dynamic distribution (B), and radar map (C).**

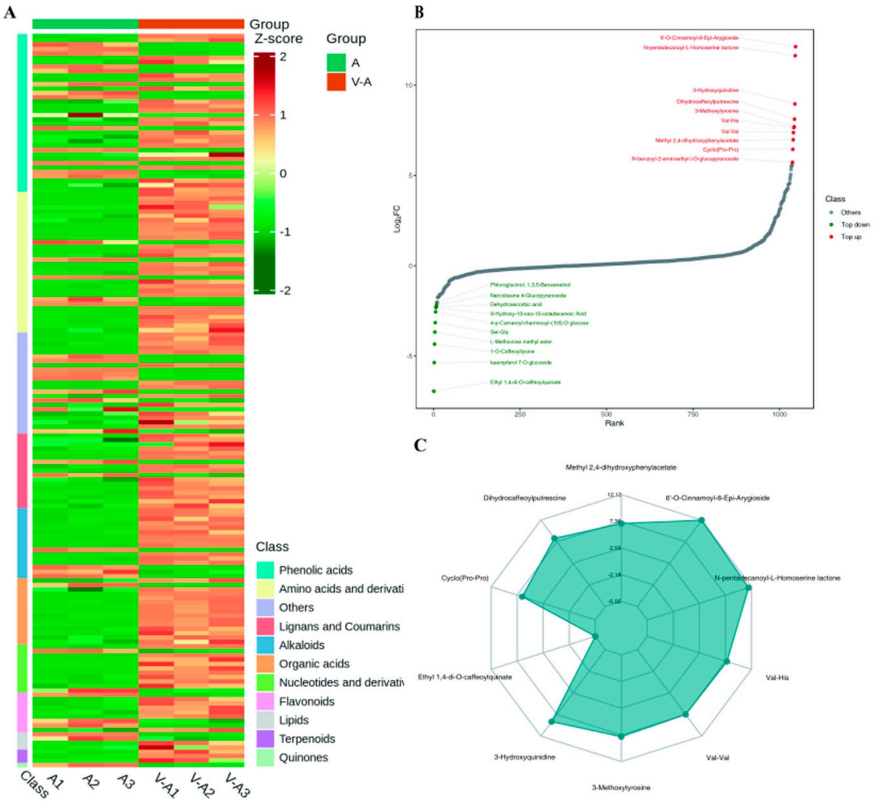

**Figure S7. Cluster heat map analysis of various differential compounds.**

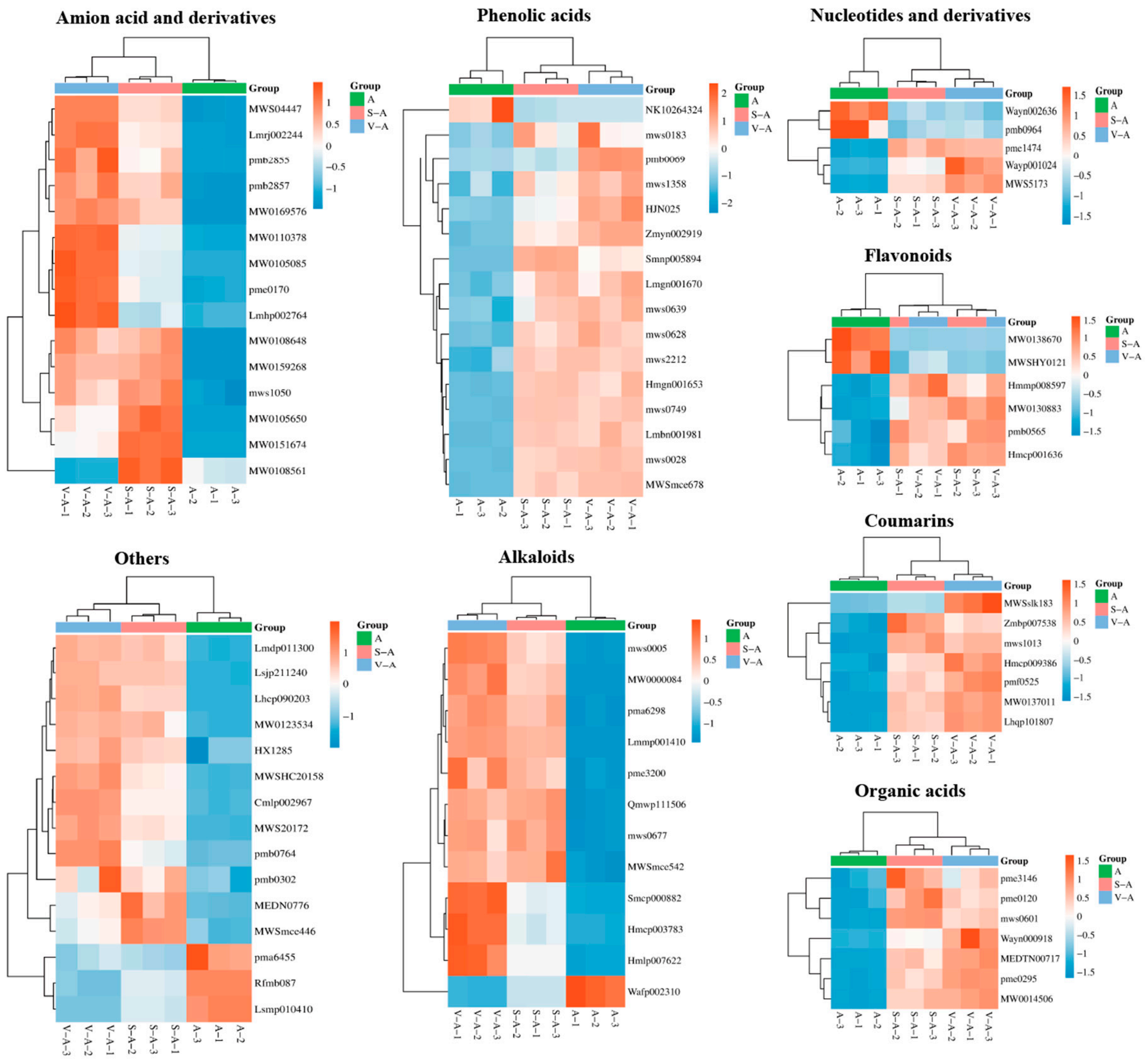

**Table S1 Sample information collection sheet. *A. Sinensis* sample number, plant origin, site of tissue, processed sample and group.**

| Number | Plant Origin | Site of Tissue | Processed sample | Group |
|---|---|---|---|---|
| A1 | *A. Sinensis* | Dried Root | *A. Sinensis* | A |
| A2 | *A. Sinensis* | Dried Root | *A. Sinensis* | A |
| A3 | *A. Sinensis* | Dried Root | *A. Sinensis* | A |
| S-A1 | *A. Sinensis* | Dried Root | Stir-fry *A. Sinensis* | S-A |
| S-A2 | *A. Sinensis* | Dried Root | Stir-fry *A. Sinensis* | S-A |
| S-A3 | *A. Sinensis* | Dried Root | Stir-fry *A. Sinensis* | S-A |
| V-A1 | *A. Sinensis* | Dried Root | Vinegar *A. Sinensis* | V-A |
| V-A2 | *A. Sinensis* | Dried Root | Vinegar *A. Sinensis* | V-A |
| V-A3 | *A. Sinensis* | Dried Root | Vinegar *A. Sinensis* | V-A |

**Table S2. 83 differential compounds of *A. sinensis* during processing.**

| Num. | Index | Formula | Compounds | Class I | A | S-A | V-A | VIP | P-value |
|---|---|---|---|---|---|---|---|---|---|
| 1 | Lmhp002764 | $C_{11}H_{18}N_2O_2$ | Cyclo(Proline-Leucine) | Amino acids and derivatives | $1.80 \times 10^4$ | $5.55 \times 10^4$ | $1.56 \times 10^5$ | 1.62 | $1.68 \times 10^{-6}$ |
| 2 | Lmrj002244 | $C_{10}H_{14}N_2O_2$ | Cyclo(Proline-Proline) | Amino acids and derivatives | $1.32 \times 10^4$ | $6.95 \times 10^5$ | $1.15 \times 10^6$ | 1.49 | $3.66 \times 10^{-8}$ |
| 3 | MW0105085 | $C_{10}H_{13}NO_4$ | 3-Methoxytyrosine | Amino acids and derivatives | $5.77 \times 10^3$ | $4.51 \times 10^5$ | $1.20 \times 10^6$ | 1.51 | $4.33 \times 10^{-8}$ |
| 4 | MW0105650 | $C_{10}H_{20}N_6O_4$ | Argnine-Asparagine | Amino acids and derivatives | $2.13 \times 10^4$ | $1.75 \times 10^6$ | $9.71 \times 10^5$ | 1.30 | $1.07 \times 10^{-6}$ |
| 5 | MW0108561 | $C_9H_{18}N_4O_4$ | *N*2-(1-Carboxyethyl)-L-arginine | Amino acids and derivatives | $3.26 \times 10^5$ | $7.54 \times 10^5$ | $8.01 \times 10^4$ | 1.01 | $5.02 \times 10^{-7}$ |
| 6 | MW0108648 | $C_{13}H_{17}NO_4$ | Ethyl *N*-acetyl-L-tyrosinate | Amino acids and derivatives | $2.02 \times 10^4$ | $6.20 \times 10^5$ | $6.14 \times 10^5$ | 1.41 | $5.77 \times 10^{-5}$ |
| 7 | MW0110378 | $C_{10}H_{20}N_2O_3$ | Valine-Valine | Amino acids and derivatives | $1.78 \times 10^3$ | $1.24 \times 10^5$ | $2.95 \times 10^5$ | 1.51 | $8.32 \times 10^{-11}$ |
| 8 | MW0151674 | $C_{12}H_{24}N_2O_3$ | Isoleucine-Leucine | Amino acids and derivatives | $4.03 \times 10^3$ | $3.58 \times 10^5$ | $1.84 \times 10^5$ | 1.29 | $4.79 \times 10^{-9}$ |
| 9 | MW0159268 | $C_{11}H_{18}N_4O_3$ | Valine-Histidine | Amino acids and derivatives | $3.26 \times 10^4$ | $7.07 \times 10^6$ | $6.54 \times 10^6$ | 1.41 | $8.25 \times 10^{-7}$ |
| 10 | MW0169576 | $C_{19}H_{35}NO_3$ | *N*-pentadecanoyl-L-Homoserine lactone | Amino acids and derivatives | 9.00 | $2.26 \times 10^4$ | $2.86 \times 10^4$ | 1.44 | $5.04 \times 10^{-8}$ |
| 11 | MWS04447 | $C_6H_{11}NO_2$ | Homoproline | Amino acids and derivatives | $5.71 \times 10^5$ | $1.66 \times 10^6$ | $2.17 \times 10^6$ | 1.55 | $4.95 \times 10^{-10}$ |
| 12 | mws1050 | $C_5H_9NO_4$ | *O*-Acetylserine | Amino acids and derivatives | $1.77 \times 10^5$ | $6.40 \times 10^5$ | $5.47 \times 10^5$ | 1.29 | $2.24 \times 10^{-5}$ |
| 13 | pmb2855 | $C_{11}H_{20}N_2O_8$ | L-Glutamine-*O*-glycoside | Amino acids and derivatives | $5.47 \times 10^4$ | $8.20 \times 10^5$ | $1.31 \times 10^6$ | 1.51 | $1.13 \times 10^{-4}$ |

| | | | | | | | | | |
|---|---|---|---|---|---|---|---|---|---|
| 14 | pmb2857 | $C_{11}H_{19}NO_9$ | L-Glutamic acid-*O*-glycoside | Amino acids and derivatives | $1.35 \times 10^4$ | $2.00 \times 10^5$ | $2.46 \times 10^5$ | 1.46 | $1.19 \times 10^{-5}$ |
| 15 | pme0170 | $C_8H_{16}N_4O_3$ | *N*-Acetyl-L-Arginine | Amino acids and derivatives | $1.01 \times 10^5$ | $2.67 \times 10^5$ | $4.99 \times 10^5$ | 1.62 | $7.48\times 10^{-7}$ |
| 16 | HJN025 | $C_7H_8O_2$ | 4-Hydroxybenzyl Alcohol | Phenolic acids | $2.32 \times 10^3$ | $2.55 \times 10^4$ | $5.34 \times 10^4$ | 1.49 | $1.65 \times 10^{-5}$ |
| 17 | Hmgn001653 | $C_7H_6O_3$ | Protocatechualdehyde | Phenolic acids | $1.78 \times 10^5$ | $1.71 \times 10^6$ | $1.63 \times 10^6$ | 1.40 | $1.12 \times 10^{-5}$ |
| 18 | Lmbn001981 | $C_7H_6O_3$ | 2,5-Dihydroxybenzaldehyde | Phenolic acids | $2.49 \times 10^5$ | $2.18 \times 10^6$ | $2.20 \times 10^6$ | 1.41 | $8.16 \times 10^{-6}$ |
| 19 | Lmgn001670 | $C_7H_6O_3$ | Salicylic acid | Phenolic acids | $1.01 \times 10^5$ | $2.20 \times 10^5$ | $2.23 \times 10^5$ | 1.36 | $2.36 \times 10^{-3}$ |
| 20 | mws0028 | $C_8H_8O_4$ | Vanillic acid | Phenolic acids | $2.92 \times 10^5$ | $1.78 \times 10^6$ | $1.92 \times 10^6$ | 1.45 | $1.34 \times 10^{-7}$ |
| 21 | mws0183 | $C_7H_6O_4$ | 3,4-Dihydroxybenzoic acid (Protocatechuic acid)* | Phenolic acids | $2.13 \times 10^5$ | $8.96 \times 10^5$ | $9.81 \times 10^5$ | 1.31 | $6.56 \times 10^{-2}$ |
| 22 | mws0628 | $C_7H_6O_2$ | 4-Hydroxybenzaldehyde | Phenolic acids | $4.09 \times 10^5$ | $8.60 \times 10^5$ | $9.27 \times 10^5$ | 1.46 | $2.93 \times 10^{-5}$ |
| 23 | mws0639 | $C_7H_6O_4$ | 2,3-Dihydroxybenzoic Acid* | Phenolic acids | $1.89 \times 10^5$ | $5.09 \times 10^5$ | $5.96 \times 10^5$ | 1.40 | $9.82 \times 10^{-4}$ |
| 24 | mws0749 | $C_7H_6O_3$ | 4-Hydroxybenzoic acid | Phenolic acids | $4.48 \times 10^5$ | $2.44 \times 10^6$ | $2.35 \times 10^6$ | 1.40 | $3.26 \times 10^{-7}$ |
| 25 | mws1358 | $C_6H_6O_2$ | Pyrocatechol | Phenolic acids | $2.09 \times 10^4$ | $4.38 \times 10^4$ | $5.42 \times 10^4$ | 1.41 | $3.10 \times 10^{-3}$ |
| 26 | mws2212 | $C_9H_8O_4$ | Caffeic acid | Phenolic acids | $2.73 \times 10^6$ | $1.06 \times 10^7$ | $1.06 \times 10^7$ | 1.32 | $1.46 \times 10^{-4}$ |
| 27 | MWSmce678 | $C_{11}H_{14}O_3$ | Deoxyarbutin | Phenolic acids | $6.37 \times 10^4$ | $2.36 \times 10^5$ | $2.63 \times 10^5$ | 1.47 | $2.22 \times 10^{-7}$ |
| 28 | NK10264324 | $C_6H_6O_3$ | Phloroglucinol; 1,3,5-Benzenetriol | Phenolic acids | $1.32 \times 10^6$ | $2.37 \times 10^5$ | $3.21 \times 10^5$ | 1.18 | $2.44 \times 10^{-2}$ |
| 29 | pmb0069 | $C_7H_7NO$ | Benzamide | Phenolic acids | $2.54 \times 10^4$ | $5.16 \times 10^4$ | $1.97 \times 10^5$ | 1.60 | $9.83 \times 10^{-8}$ |
| 30 | Smnp005894 | $C_{25}H_{28}O_{12}$ | 6'-*O*-Cinnamoyl-8-Epi-Arygioside | Phenolic acids | 9.00 | $6.49 \times 10^4$ | $4.04 \times 10^4$ | 1.38 | $5.08 \times 10^{-7}$ |
| 31 | Zmyn002919 | $C_8H_8O_2$ | 2-Methylbenzoic acid | Phenolic acids | $2.07 \times 10^3$ | $2.35 \times 10^4$ | $3.46 \times 10^4$ | 1.48 | $5.30 \times 10^{-7}$ |
| 32 | MWS5173 | $C_4H_5N_3O$ | Isocytosine | Nucleotides and derivatives | $1.02 \times 10^6$ | $2.80 \times 10^6$ | $3.50 \times 10^6$ | 1.53 | $7.62 \times 10^{-8}$ |
| 33 | pmb0964 | $C_{16}H_{23}N_5O_5$ | Isopentenyladenine-7-N-glucoside | Nucleotides and derivatives | $7.06 \times 10^5$ | $2.17 \times 10^5$ | $2.54 \times 10^5$ | 1.14 | $1.21 \times 10^{-2}$ |
| 34 | pme1474 | $C_{11}H_{15}N_5O_3S$ | 5'-Deoxy-5'-(methylthio)adenosine | Nucleotides and derivatives | $1.19 \times 10^7$ | $4.08 \times 10^7$ | $3.97 \times 10^7$ | 1.40 | $1.82 \times 10^{-6}$ |
| 35 | Wayn002636 | $C_{17}H_{22}N_2O_7$ | *N*-(1-Deoxy-1-fructosyl)Tryptophan | Nucleotides and derivatives | $2.57 \times 10^6$ | $1.21 \times 10^6$ | $1.13 \times 10^6$ | 1.44 | $2.62 \times 10^{-5}$ |

| | | | | | | | | | |
|---|---|---|---|---|---|---|---|---|---|
| 36 | Wayp001024 | $C_{11}H_{21}NO_7$ | *N*-(1-Deoxy-1-fructosyl)Valine | Nucleotides and derivatives | $1.03 \times 10^4$ | $2.47 \times 10^5$ | $4.89 \times 10^5$ | 1.53 | $4.60 \times 10^{-5}$ |
| 37 | Hmcp001636 | $C_{23}H_{24}O_{13}$ | Limocitrin-7-*O*-glucoside | Flavonoids | $7.72 \times 10^4$ | $1.82 \times 10^5$ | $1.67 \times 10^5$ | 1.31 | $3.76 \times 10^{-5}$ |
| 38 | Hmmp008597 | $C_{16}H_{14}O_4$ | 4,4'-Dihydroxy-2'-methoxychalcone (3-Deoxysappanchalcone) | Flavonoids | $1.04 \times 10^4$ | $4.99 \times 10^4$ | $6.57 \times 10^4$ | 1.48 | $5.58 \times 10^{-5}$ |
| 39 | MW0130883 | $C_{19}H_{18}O_5$ | 2-Methyl-5,7,8-trimethoxyisoflavone | Flavonoids | $4.46 \times 10^4$ | $1.16 \times 10^5$ | $1.23 \times 10^5$ | 1.43 | $9.92 \times 10^{-4}$ |
| 40 | MW0138670 | $C_{21}H_{20}O_{11}$ | kaempferol 7-*O*-glucoside | Flavonoids | $1.08 \times 10^6$ | $3.60 \times 10^4$ | $2.62 \times 10^4$ | 1.47 | $3.80 \times 10^{-6}$ |
| 41 | MWSHY0121 | $C_{21}H_{18}O_{12}$ | Luteolin-7-*O*-glucuronide | Flavonoids | $7.43 \times 10^4$ | $2.67 \times 10^4$ | $3.33 \times 10^4$ | 1.18 | $1.75 \times 10^{-4}$ |
| 42 | pmb0565 | $C_{23}H_{24}O_{13}$ | Syringetin-3-*O*-glucoside | Flavonoids | $7.08 \times 10^4$ | $1.59 \times 10^5$ | $1.58 \times 10^5$ | 1.35 | $5.52 \times 10^{-4}$ |
| 43 | Hmcp009386 | $C_{16}H_{14}O_4$ | Alloimperatorin | Lignans and Coumarins | $1.04 \times 10^4$ | $4.89 \times 10^4$ | $6.52 \times 10^4$ | 1.48 | $7.28 \times 10^{-6}$ |
| 44 | HX1285 | $C_{12}H_{14}O_3$ | 4-(3-Methoxy-4-hydroxy)pheny-3-methy1-3-buten-2-one | Lignans and Coumarins | $1.63 \times 10^5$ | $4.50 \times 10^5$ | $5.09 \times 10^5$ | 1.18 | $1.29 \times 10^{-3}$ |
| 45 | Lhqp101807 | $C_{15}H_{16}O_3$ | Osthole | Lignans and Coumarins | $8.76 \times 10^5$ | $9.72 \times 10^6$ | $1.21 \times 10^7$ | 1.47 | $1.08 \times 10^{-8}$ |
| 46 | MW0137011 | $C_{17}H_{14}O_4$ | 7-Hydroxy-3-(4-methoxyphenyl)-4-methylcoumarin | Lignans and Coumarins | $9.00 \times 10^5$ | $7.97 \times 10^6$ | $9.47 \times 10^6$ | 1.47 | $5.57 \times 10^{-8}$ |
| 47 | mws1013 | $C_9H_6O_4$ | Esculetin (6,7-Dihydroxycoumarin) | Lignans and Coumarins | $2.55 \times 10^5$ | $8.01 \times 10^5$ | $7.49 \times 10^5$ | 1.37 | $1.45 \times 10^{-6}$ |
| 48 | MWSslk183 | $C_{15}H_{10}O_4$ | 7,8-Dihydroxy-4-phenylcoumarin | Lignans and Coumarins | $1.16 \times 10^4$ | $3.19 \times 10^4$ | $1.27 \times 10^5$ | 1.62 | $9.30 \times 10^{-6}$ |
| 49 | pmf0525 | $C_{16}H_{14}O_4$ | Imperatorin | Lignans and Coumarins | $6.90 \times 10^3$ | $5.90 \times 10^4$ | $7.79 \times 10^4$ | 1.46 | $4.76 \times 10^{-6}$ |
| 50 | Zmbp007538 | $C_{14}H_{14}O_3$ | Osthenol | Lignans and Coumarins | $8.33 \times 10^4$ | $2.13 \times 10^5$ | $1.75 \times 10^5$ | 1.21 | $2.82 \times 10^{-5}$ |
| 51 | Hmcp003783 | $C_{13}H_{10}N_2O$ | 1-Acetyl-β-carboline | Alkaloids | $1.35 \times 10^4$ | $1.19 \times 10^5$ | $3.03 \times 10^5$ | 1.59 | $5.84 \times 10^{-8}$ |
| 52 | Hmlp007622 | $C_{18}H_{29}NO_3$ | Decanoyl vanillylamide | Alkaloids | $6.70 \times 10^6$ | $1.35 \times 10^7$ | $2.13 \times 10^7$ | 1.62 | $2.31 \times 10^{-6}$ |
| 53 | Lmmp001410 | $C_{13}H_{20}N_2O_3$ | Dihydrocaffeoylputrescine | Alkaloids | $7.41 \times 10^3$ | $1.84 \times 10^6$ | $2.06 \times 10^6$ | 1.43 | $2.84 \times 10^{-12}$ |
| 54 | MW0000084 | $C_{20}H_{24}N_2O_3$ | 3-Hydroxyquinidine | Alkaloids | $4.58 \times 10^3$ | $1.74 \times 10^6$ | $2.28 \times 10^6$ | 1.44 | $4.54 \times 10^{-7}$ |

| | | | | | | | | | |
|---|---|---|---|---|---|---|---|---|---|
| 55 | mws0005 | $C_{10}H_{12}N_2$ | Tryptamine | Alkaloids | $8.49 \times 10^4$ | $7.27 \times 10^5$ | $9.96 \times 10^5$ | 1.50 | $2.54 \times 10^{-8}$ |
| 56 | mws0677 | $C_{12}H_{14}N_2O_2$ | N-Acetyl-5-hydroxytryptamine | Alkaloids | $4.21 \times 10^5$ | $1.28 \times 10^6$ | $1.21 \times 10^6$ | 1.36 | $2.89 \times 10^{-5}$ |
| 57 | MWSmce542 | $C_{12}H_{14}N_2O_2$ | (S)-Indoximod | Alkaloids | $1.07 \times 10^5$ | $3.04 \times 10^5$ | $2.79 \times 10^5$ | 1.34 | $3.34 \times 10^{-5}$ |
| 58 | pma6298 | $C_5H_5NO$ | 3-Hydroxypyridine | Alkaloids | $1.94 \times 10^4$ | $2.97 \times 10^5$ | $3.40 \times 10^5$ | 1.44 | $5.49 \times 10^{-7}$ |
| 59 | pme3200 | $C_2H_7N_3$ | 1-Methylguanidine | Alkaloids | $1.31 \times 10^4$ | $1.30 \times 10^5$ | $1.57 \times 10^5$ | 1.42 | $1.11 \times 10^{-4}$ |
| 60 | Qmwp111506 | $C_{21}H_{29}NO$ | 1-Methyl-2-[(Z)-6-undecyl]-4(1H)-quinolone | Alkaloids | $2.25 \times 10^4$ | $8.10 \times 10^4$ | $7.74 \times 10^4$ | 1.39 | $8.37\times 10^{-7}$ |
| 61 | Smcp000882 | $C_{15}H_{21}NO_7$ | N-benzoyl-2-aminoethyl-β-D-glucopyranoside | Alkaloids | $1.08 \times 10^4$ | $2.41 \times 10^5$ | $5.70 \times 10^5$ | 1.55 | $1.99 \times 10^{-8}$ |
| 62 | Wafp002310 | $C_{15}H_{20}N_2O_5$ | 1-O-Caffeoyllysine | Alkaloids | $1.83 \times 10^6$ | $5.97 \times 10^5$ | $9.05 \times 10^4$ | 1.61 | $3.30 \times 10^{-7}$ |
| 63 | MEDTN00717 | $C_{20}H_{26}O_3$ | 4-Oxoretinoic acid | Organic acids | $7.48 \times 10^3$ | $7.72 \times 10^4$ | $9.76 \times 10^4$ | 1.47 | $4.63 \times 10^{-6}$ |
| 64 | MW0014506 | $C_{20}H_{28}O_3$ | 4-Hydroxyretinoic acid | Organic acids | $3.92 \times 10^5$ | $3.91 \times 10^6$ | $4.61 \times 10^6$ | 1.46 | $2.79 \times 10^{-6}$ |
| 65 | mws0601 | $C_5H_5NO_2$ | Pyrrole-2-carboxylic acid | Organic acids | $3.08 \times 10^4$ | $1.83 \times 10^5$ | $1.47 \times 10^5$ | 1.31 | $6.80 \times 10^{-8}$ |
| 66 | pme0120 | $C_5H_{11}NO_2$ | 5-Aminovaleric acid | Organic acids | $8.62 \times 10^4$ | $2.46 \times 10^5$ | $2.07 \times 10^5$ | 1.26 | $1.42 \times 10^{-4}$ |
| 67 | pme0295 | $C_6H_{11}NO_3$ | 4-Acetamidobutyric acid | Organic acids | $2.89 \times 10^5$ | $6.13 \times 10^5$ | $7.12 \times 10^5$ | 1.52 | $1.96 \times 10^{-7}$ |
| 68 | pme3146 | $C_5H_{10}N_2O_3$ | β-Ureidoisobutyric acid | Organic acids | $3.46 \times 13$ | $1.64 \times 10^4$ | $1.18 \times 10^4$ | 1.16 | $1.14 \times 10^{-3}$ |
| 69 | Wayn000918 | $C_{18}H_{28}O_{17}$ | Isocitric acid-1-*O*-diglucoside | Organic acids | $8.48 \times 10^4$ | $3.23 \times 10^5$ | $5.61 \times 10^5$ | 1.58 | $2.08 \times 10^{-4}$ |
| 70 | Lmdp011300 | $C_{17}H_{20}O_4$ | Angeloyl Senkyunolide F | Others | $1.82 \times 10^6$ | $4.37 \times 10^6$ | $4.72 \times 10^6$ | 1.47 | $7.76 \times 10^{-7}$ |
| 71 | Lsjp211240 | $C_6H_6N_2O$ | Nicotinamide | Others | $1.59 \times 10^6$ | $2.48 \times 10^7$ | $2.71 \times 10^7$ | 1.44 | $1.02 \times 10^{-7}$ |
| 72 | Lsmp010410 | $C_{27}H_{28}O_{12}$ | Ethyl 1,4-di-*O*-caffeoylquinate | Others | $3.27 \times 10^6$ | $9.43 \times 10^5$ | $2.65 \times 10^4$ | 1.57 | $3.08 \times 10^{-7}$ |
| 73 | pmb0302 | $C_2H_8NO_3P$ | 2-Aminoethylphosphonate | Others | $1.82 \times 10^4$ | $4.25 \times 10^4$ | $4.46 \times 10^4$ | 1.20 | $5.08 \times 10^{-2}$ |
| 74 | pmb0764 | $C_6H_9NOS$ | 4-Methyl-5-thiazoleethanol | Others | $3.42 \times 10^4$ | $1.90 \times 10^5$ | $4.22 \times 10^5$ | 1.59 | $1.06 \times 10^{-6}$ |
| 75 | MWS20172 | $C_6H_6O_3$ | 5-Hydroxymethylfurfural | Others | $2.54 \times 10^5$ | $4.02 \times 10^6$ | $5.93 \times 10^6$ | 1.50 | $1.32 \times 10^{-7}$ |
| 76 | MWSHC20158 | $C_6H_6O_3$ | 5-Methoxyfurfural | Others | $3.03 \times 10^5$ | $3.66 \times 10^6$ | $5.84 \times 10^6$ | 1.51 | $4.84 \times 10^{-7}$ |
| 77 | Lhcp090203 | $C_{13}H_{20}O$ | α-Ionone | Others | $4.41 \times 10^5$ | $2.57 \times 10^6$ | $3.20 \times 10^6$ | 1.49 | $3.19 \times 10^{-9}$ |
| 78 | MW0123534 | $C_{10}H_{18}O_2$ | delta-Decalactone | Others | $6.04 \times 10^5$ | $3.72 \times 10^6$ | $4.22 \times 10^6$ | 1.46 | $1.24 \times 10^{-4}$ |
| 79 | MWSmce446 | $C_{22}H_{28}O_{11}$ | Cimifugin-7-*O*-glucoside | Others | $2.02 \times 10^4$ | $1.35 \times 10^5$ | $6.95 \times 10^4$ | 1.02 | $3.73 \times 10^{-5}$ |
| 80 | MEDN0776 | $C_{20}H_{32}O_4$ | 5,15-DiHETE | Lipids | $1.85 \times 10^5$ | $2.96 \times 10^6$ | $1.63 \times 10^6$ | 1.21 | $3.44 \times 10^{-4}$ |
| 81 | Rfmb087 | $C_{18}H_{32}O_4$ | 9-Hydroxy-13-oxo-10-octadecenoic | Lipids | $4.94 \times 10^5$ | $2.23 \times 10^5$ | $9.95 \times 10^4$ | 1.62 | $3.72 \times 10^{-8}$ |

| | | | | | | | | | |
|---|---|---|---|---|---|---|---|---|---|
| | | | Acid | | | | | | |
| 82 | pma6455 | $C_5H_{11}O_8P$ | Ribulose-5-phosphate | Others | $2.53 \times 10^5$ | $1.24 \times 10^5$ | $1.25 \times 10^5$ | 1.34 | $1.12 \times 10^{-3}$ |
| 83 | Cmlp002967 | $C_{10}H_{12}O_5$ | Gentiolactone | Terpenoids | $8.90 \times 10^3$ | $7.69 \times 10^4$ | $1.29 \times 10^5$ | 1.53 | $2.69 \times 10^{-9}$ |

**Table S3. Grey correlation degree analysis. Grey correlation degree analysis between differential components and antioxidant activity of A. sinensis and its processed products.**

| Number | DPPH free radical scavenging | | Hydroxyl free radical scavenging | | T-AOC | |
|---|---|---|---|---|---|---|
| | Chemical composition | Correlation degree | Chemical composition | Correlation degree | Chemical composition | Correlation degree |
| 1 | mws0628 | 0.857 | mws0628 | 0.814 | mws0628 | 0.851 |
| 2 | pmb0565 | 0.856 | pme0295 | 0.81 | pme0295 | 0.844 |
| 3 | Hmcp001636 | 0.849 | pmb0565 | 0.809 | Hmcp001636 | 0.843 |
| 4 | pme0295 | 0.847 | Hmcp001636 | 0.805 | pmb0565 | 0.84 |
| 5 | mws1358 | 0.847 | Lmgn001670 | 0.805 | Zmbp007538 | 0.838 |
| 6 | Lmgn001670 | 0.84 | Zmbp007538 | 0.801 | Lmgn001670 | 0.835 |
| 7 | Hmlp007622 | 0.836 | pmb0302 | 0.793 | Lmdp011300 | 0.829 |
| 8 | Zmbp007538 | 0.835 | Lmdp011300 | 0.793 | pme0120 | 0.821 |
| 9 | Lmdp011300 | 0.834 | mws1358 | 0.792 | mws1358 | 0.819 |
| 10 | pme0120 | 0.827 | pma6455 | 0.786 | pmb0302 | 0.815 |
| 11 | MW0130883 | 0.825 | pme0120 | 0.785 | MWSmce542 | 0.815 |
| 12 | MWSmce542 | 0.821 | MWSmce542 | 0.779 | HX1285 | 0.813 |
| 13 | HX1285 | 0.816 | HX1285 | 0.779 | MWS5173 | 0.808 |
| 14 | pmb0302 | 0.813 | MWS5173 | 0.774 | mws0677 | 0.804 |
| 15 | mws1013 | 0.812 | mws0677 | 0.769 | MWS04447 | 0.802 |
| 16 | mws0639 | 0.811 | MWS04447 | 0.769 | mws1013 | 0.8 |
| 17 | MWS5173 | 0.805 | MW0130883 | 0.768 | MW0130883 | 0.799 |
| 18 | mws0677 | 0.802 | mws1013 | 0.765 | mws1050 | 0.79 |
| 19 | pme1474 | 0.798 | Wayn002636 | 0.763 | pme1474 | 0.788 |
| 20 | MWS04447 | 0.797 | Hmlp007622 | 0.761 | Qmwp111506 | 0.783 |
| 21 | mws1050 | 0.796 | mws1050 | 0.755 | mws0639 | 0.783 |
| 22 | Qmwp111506 | 0.794 | mws0639 | 0.754 | Hmlp007622 | 0.779 |
| 23 | mws2212 | 0.784 | pme1474 | 0.754 | mws2212 | 0.777 |
| 24 | MWSmce678 | 0.779 | Qmwp111506 | 0.75 | MWSmce678 | 0.777 |
| 25 | Wayn000918 | 0.776 | MWSmce678 | 0.744 | mws0183 | 0.765 |
| 26 | pme0170 | 0.773 | MWSHY0121 | 0.743 | pme3146 | 0.764 |

| | | | | | | |
|---|---|---|---|---|---|---|
| 27 | mws0601 | 0.767 | mws2212 | 0.743 | pma6455 | 0.76 |
| 28 | pme3146 | 0.76 | pme3146 | 0.738 | Hmcp009386 | 0.757 |
| 29 | mws0749 | 0.757 | mws0183 | 0.736 | mws0601 | 0.749 |
| 30 | Hmmp008597 | 0.757 | Hmcp009386 | 0.732 | Hmmp008597 | 0.748 |
| 31 | Hmcp009386 | 0.756 | Hmmp008597 | 0.724 | mws0749 | 0.748 |
| 32 | pma6455 | 0.754 | Wayn000918 | 0.721 | Lhcp090203 | 0.745 |
| 33 | mws0183 | 0.749 | MWSmce446 | 0.72 | MWSmce446 | 0.744 |
| 34 | MW0123534 | 0.747 | pmb0964 | 0.72 | mws0028 | 0.74 |
| 35 | mws0028 | 0.744 | mws0601 | 0.716 | Wayn002636 | 0.739 |
| 36 | Lhcp090203 | 0.742 | mws0749 | 0.716 | Wayn000918 | 0.734 |
| 37 | Wayn002636 | 0.736 | Lhcp090203 | 0.714 | mws0005 | 0.733 |
| 38 | Cmlp002967 | 0.732 | Cmlp002967 | 0.714 | pmf0525 | 0.732 |
| 39 | Lmbn001981 | 0.731 | pme0170 | 0.712 | Zmyn002919 | 0.728 |
| 40 | Hmgn001653 | 0.73 | mws0028 | 0.709 | pme0170 | 0.727 |
| 41 | pmf0525 | 0.726 | mws0005 | 0.703 | MW0123534 | 0.727 |
| 42 | mws0005 | 0.725 | pmf0525 | 0.702 | Cmlp002967 | 0.727 |
| 43 | pme3200 | 0.724 | MWSHC2015<br>8 | 0.701 | pme3200 | 0.725 |
| 44 | MW0137011 | 0.724 | MW0123534 | 0.701 | MW0137011 | 0.724 |
| 45 | pmb2855 | 0.722 | Zmyn002919 | 0.701 | Lmbn001981 | 0.724 |
| 46 | MWSHC2015<br>8 | 0.722 | MW0108561 | 0.698 | MWSHC2015<br>8 | 0.722 |
| 47 | MW0014506 | 0.72 | Rfmb087 | 0.697 | MWS20172 | 0.722 |
| 48 | HJN025 | 0.72 | pme3200 | 0.696 | MW0014506 | 0.721 |
| 49 | MEDTN00717 | 0.72 | MEDTN00717 | 0.695 | MWSHY0121 | 0.72 |
| 50 | Zmyn002919 | 0.719 | MW0137011 | 0.694 | Hmgn001653 | 0.72 |
| 51 | pmb0764 | 0.717 | MWS20172 | 0.693 | MEDTN00717 | 0.719 |
| 52 | Lhqp101807 | 0.716 | Lmbn001981 | 0.693 | Lhqp101807 | 0.719 |
| 53 | Wayp001024 | 0.713 | MW0014506 | 0.691 | MW0151674 | 0.713 |

| | | | | | | |
|---|---|---|---|---|---|---|
| 54 | MWS20172 | 0.712 | Hmgn001653 | 0.69 | pmb2857 | 0.713 |
| 55 | MWSHY0121 | 0.711 | Lmrj002244 | 0.689 | Lmrj002244 | 0.712 |
| 56 | pmb2857 | 0.71 | Lhqp101807 | 0.689 | MW0105650 | 0.712 |
| 57 | pma6298 | 0.709 | MEDN0776 | 0.688 | MEDN0776 | 0.712 |
| 58 | Lsjp211240 | 0.708 | MW0151674 | 0.686 | pma6298 | 0.708 |
| 59 | MWSmce446 | 0.708 | pmb2855 | 0.686 | MW0108561 | 0.708 |
| 60 | Lmrj002244 | 0.706 | pmb2857 | 0.684 | Lsjp211240 | 0.705 |
| 61 | MW0108648 | 0.705 | MW0105650 | 0.681 | pmb2855 | 0.703 |
| 62 | MEDN0776 | 0.704 | pma6298 | 0.679 | pmb0964 | 0.698 |
| 63 | Smcp000882 | 0.698 | Lsjp211240 | 0.676 | Smnp005894 | 0.697 |
| 64 | Smnp005894 | 0.697 | Wayp001024 | 0.67 | MW0108648 | 0.697 |
| 65 | MW0110378 | 0.693 | HJN025 | 0.668 | MW0000084 | 0.694 |
| 66 | MW0159268 | 0.692 | MW0108648 | 0.668 | MW0169576 | 0.692 |
| 67 | Lmhp002764 | 0.692 | Smnp005894 | 0.666 | Wayp001024 | 0.687 |
| 68 | Hmcp003783 | 0.69 | MW0000084 | 0.666 | Lmmp001410 | 0.685 |
| 69 | pmb0964 | 0.69 | MW0169576 | 0.664 | MW0159268 | 0.683 |
| 70 | MW0000084 | 0.689 | pmb0764 | 0.662 | Rfmb087 | 0.68 |
| 71 | MW0105650 | 0.689 | Lmmp001410 | 0.657 | HJN025 | 0.68 |
| 72 | Rfmb087 | 0.688 | MW0159268 | 0.654 | pmb0764 | 0.675 |
| 73 | MW0169576 | 0.688 | NK10264324 | 0.651 | Smcp000882 | 0.652 |
| 74 | Lmmp001410 | 0.687 | Smcp000882 | 0.64 | Lmhp002764 | 0.65 |
| 75 | MW0108561 | 0.684 | MW0110378 | 0.635 | MW0110378 | 0.647 |
| 76 | MW0151674 | 0.68 | Lmhp002764 | 0.634 | Hmcp003783 | 0.646 |
| 77 | MW0105085 | 0.673 | Hmcp003783 | 0.633 | NK10264324 | 0.636 |
| 78 | pmb0069 | 0.658 | MW0105085 | 0.619 | MW0105085 | 0.631 |
| 79 | MWSslk183 | 0.643 | Wafp002310 | 0.618 | pmb0069 | 0.622 |
| 80 | NK10264324 | 0.629 | pmb0069 | 0.605 | MWSslk183 | 0.608 |
| 81 | Wafp002310 | 0.616 | Lsmp010410 | 0.594 | Wafp002310 | 0.607 |

| | | | | | | |
|---|---|---|---|---|---|---|
| 82 | Lsmp010410 | 0.594 | MWSslk183 | 0.592 | Lsmp010410 | 0.585 |
| 83 | MW0138670 | 0.5 | MW0138670 | 0.502 | MW0138670 | 0.502 |
